# How water quality affects perceived risk of waterborne disease: evidence from Zapopan, Mexico

**DOI:** 10.1186/s13690-026-01847-w

**Published:** 2026-02-06

**Authors:** Alejandro Lome-Hurtado, Pia Berger, Hugo Briseno

**Affiliations:** 1 Facultad de Ciencias Económicas y Empresariales, Universidad Panamericana, Álvaro del Portillo 49, Zapopan, 45010 Jalisco Mexico; 2 Facultad de Ingeniería, Universidad Panamericana, Jose María Escrivá de Balaguer 101, Villas Bonaterra, Aguascalientes, 20296 Aguascalientes Mexico

**Keywords:** Water quality, Water insecurity, Public health equity, Environmental exposure

## Abstract

**Background:**

Access to safe and clean drinking water is a key determinant of public health and well-being. This study explores the relationship between perceived unpleasant odor of tap water (as a proxy of water quality) and perception of health risks from tap water, in low- and middle-income areas of Mexico, emphasizing the role of perceptions shaped by sensory indicators such as taste, color, and odor. The connection between perceived unpleasant odor of tap water and perceived risk warrants attention as a public health concern, given its potential to influence consumer behavior, risk exposure, and trust in health-related products and services.

**Methods:**

The analysis draws on household survey data collected through face-to-face interviews in Zapopan, Jalisco, Mexico, in October 2018, complemented with a review of existing literature. Statistical regression models were executed to assess the association between perceived water quality and perceived health risk from drinking tap water.

**Results:**

The analysis shows that households perceiving an unpleasant odor in tap water had 29% higher odds of identifying it as a health risk (OR = 1.29), controlling for socioeconomic and demographic conditions.

**Conclusions:**

The findings underscore the need for public health interventions that address both actual and perceived water quality. Integrated water policies should prioritize equity, strengthen community trust, and incorporate engagement strategies to mitigate health risks linked to water insecurity.

**Table:** 

Text box 1. Contributions to the literature
• This study provides novel empirical evidence from Mexico by examining the association between perceived unpleasant odor of tap water and perceived health risk from drinking tap water, controlling for key socioeconomic and demographic factors.
• The findings show that perceived poor quality of tap water, particularly unpleasant odor, is associated with a higher likelihood of identifying tap water as a health risk, even after adjusting for socioeconomic and demographic characteristics.
• By highlighting how perceptions related to tap water use are associated with perceived health risk, this study underscores the urgent need for integrated water policies that address both objective water quality and public perceptions, strengthen trust in municipal tap water systems, and reduce unnecessary reliance on bottled water.

## Background

Access to safe and clean drinking water is a fundamental pillar of public health and a recognized human right. Globally, inadequate water quality contributes significantly to the burden of disease, particularly through the transmission of waterborne illnesses such as diarrhea, cholera, and hepatitis A [1–3]. The World Health Organization (WHO, 2019) estimates that contaminated drinking water causes over 800,000 deaths annually, primarily due to preventable gastrointestinal infections. Safe water and effective waste management are essential for preventing and controlling disease outbreaks like COVID-19, as they enable hygiene practices and reduce pathogen transmission. However, the pandemic increased water consumption by up to 40% and wastewater generation, straining water resources and infrastructure, particularly in developing countries, while concerns about water quality and sustainability have grown despite the low risk of COVID-19 transmission through sewage systems [4].

More recently, WHO (2022) reported that an estimated 829,000 people die each year from diarrhea attributable to unsafe drinking water, sanitation, and hygiene. In recognition of this critical issue, the United Nations enshrined access to clean water and sanitation in Sustainable Development Goal 6, highlighting it as essential for health and equity [5, 6].

Despite these global commitments, substantial disparities remain, particularly in low- and middle-income countries. In Mexico, while coverage of piped water is relatively high in urban areas, concerns about water quality persist [7, 8]. National surveys suggest that more than 75% of households distrust the safety of municipal tap water, with many resorting to boiling water or purchasing bottled water to mitigate perceived health risks [9]. These behaviors often arise not from confirmed contamination but from perceptions of poor water quality, especially when sensory attributes—such as taste, odor, or clarity—suggest uncleanliness [10, 11].

Indeed, perceptions of water quality are known to influence health-related decisions, sometimes even more strongly than objective indicators [12]. Prior studies have demonstrated that fear of contracting illnesses from tap water can result in significant behavioral adaptations, such as increased use of bottled water or household filtration systems (Rodríguez et al., 2023). These actions may have unintended consequences, including environmental burdens and economic inequalities. In communities along the U.S.–Mexico border, for instance, doubts about water safety led to widespread reliance on bottled water, despite a lack of evidence that it was safer than tap sources [11].

In Mexico, poor water quality remains a pressing issue, with documented exposure to microbiological and chemical contaminants in both rural and urban areas [13, 14]. Recent estimates indicate that 4.9% of households in Mexico do not have piped water, 19.3% have at some point lacked sufficient water for hygiene, and 18.9% perceive their water as unfit for human consumption [15]. Beyond physical health, such conditions undermine trust in public services and deepen social inequalities. Perceived health risks from tap water may amplify these disparities, especially when protective behaviors are limited by socioeconomic status [16].

In Mexico, tap water supplied by municipal utilities is substantially cheaper than bottled water: typical residential tariffs correspond to costs on the order of $10^{-4}$ to $10^{-3}$ USD per liter, whereas bottled water retail prices are commonly 0.5–1 USD per liter, representing a price difference of several orders of magnitude [17, 18].

While the connection between water quality and health outcomes has been explored extensively in the literature—using both observational and experimental approaches [19, 20] — there is comparatively little empirical research in Mexico that focuses on subjective health risk perception. However, there are few similar works such as [9], who investigated willingness to pay for water quality improvements in Zapopan, Jalisco. Yet, few studies have examined how perceived water quality (e.g., water that "smells bad") correlates with perceived risk of waterborne illness, particularly when accounting for household-level socioeconomic and demographic variables. This study addresses that gap by investigating the association between perceived unpleasant odor of tap water and the perceived risk of waterborne disease among households in Zapopan, Jalisco—a municipality of over 1.4 million inhabitants facing documented water-quality challenges [14]. Using binomial logistic regression on a representative survey, we assess how perceived odor in tap water interacts with factors such as income, education, home ownership, age, and water-boiling practices to influence health risk perception.

This research makes three key contributions: a) It quantifies the relationship between perceived unpleasant odor of tap water and perceived health risk from tap water, b) It identifies social determinants of risk perception, including key economic and demographic factors, and c) It provides evidence to support targeted public health messaging and interventions aimed at addressing public perceptions of tap water quality, increasing trust in public water systems, and reducing unnecessary reliance on bottled water. By focusing on subjective perceptions as a determinant of health-related behavior, this study contributes to a deeper understanding of how environmental factors shape individual and community-level health outcomes. These findings are directly relevant to equity-driven public health strategies and sustainable urban water governance in Mexico and comparable contexts.

## Data and methods

### Area of study, water, health, and socioeconomic data

Zapopan, City, Mexico is the municipality of Jalisco with the largest population, and the seventh at the national level with a 1 million 476 thousand 491 inhabitants [21]. Zapopan, Jalisco, is one of the most economically significant municipalities in Mexico. It holds the 10th position nationwide in terms of GDP and ranks second within Jalisco, following Guadalajara [22, 23]. The choice of the case study of Zapopan, City, Mexico is consistent with growing concerns about health and its relationship with quality of water. Potable water in Zapopan is supplied primarily through three sources: the Chapala Lake aqueduct (61%), groundwater wells (27%), and the Calderón Dam (12%) [24]. However, water contamination remains a major concern in Zapopan, driven by agricultural, industrial, and urban discharges into local basins, while limited treatment infrastructure fails to remove all pollutants [9, 14, 16]. As a result, there is a public distrust toward tap water, leading most households to rely on bottled water for daily consumption. Nearly all respondents in Zapopan report purchasing bottled water as their main drinking source [25]. Note that this pattern of distrust is not unique to Zapopan. In Mexico City, for example, long-standing concerns about contamination have led to distrust of tap-water, resulting in one of the highest levels of bottled-water consumption in nationwide [26]. Similarly, recent studies in Monterrey indicate that water-supply disruptions and low trust in tap-water quality have contributed to increased bottled-water use [27]. These cases highlight that, although local water infrastructure may differ between regions, distrust in tap water remains a common driver of bottled-water dependence in Mexican cities.

In Zapopan, the water infrastructure consists of 43 water intake points for public supply, 33 of which are wells and 10 are springs. However, only two are currently operating, three are out of service, and 38 lack macrometers, making it difficult to control the resource and plan the service [21]. Moreover, the municipality does not have complete information about the number of absorption wells, although it acknowledges their existence in some neighborhoods and states that maintenance is performed when necessary. Despite achieving 97.21% coverage of potable water in 2020, deficiencies in service quality and supply shortages persist, such as those recorded in 2021 due to low water levels in the Calderón Dam, which affected neighborhoods of different socioeconomic levels. An study found that 69% of households believe the supplied water is contaminated, 53% report an unpleasant odor in tap water, and 74% fear for their health when drinking tap water; therefore, the majority of the surveyed households, 99% , reported purchasing bottled water [25]. This situation highlights the need to strengthen hydraulic infrastructure, ensure proper maintenance of distribution systems, and improve water quality to guarantee the human right to safe access to water in the municipality [28]. Consequently, distrust in the quality of tap water remains widespread among residents. In fact, a local survey data confirm that this perception strongly influences household practices: 99% of surveyed households reported drinking bottled water, 94% boiled tap water before consumption, and 74% expressed concern about becoming ill from it [25] . These figures highlight how environmental conditions and perceived risks shape everyday water use behaviors in Zapopan.

Water, health, and socioeconomic data were obtained from a previous household survey on water consumption conducted in Zapopan City, Jalisco, Mexico [25]. The household survey was implemented through face-to-face interviews conducted by trained enumerators during October 2018 in 20 neighborhoods across the municipality. These neighborhoods were selected to represent different socioeconomic strata. All survey items were closed-ended questions designed to assess household perceptions of water quality, perceived health risk, and related water-use practices, following formats commonly used in previous studies [29, 30].

The municipality of Zapopan had a total of 277,657 households in 2018 [31]. Based on this population size, a minimum sample of 384 households is statistically representative with a 95% confidence level and a 5% margin of error [25]. The survey comprised a final sample of 400 households, exceeding this minimum requirement. The sampling strategy followed a socioeconomic stratification based on the classification of the Mexican Association of Market Intelligence and Opinion Agencies (AMAI). Specifically, 30% of the surveyed households belonged to the upper and upper-middle income strata (ABC+), 35% to the middle-income stratum (C), and 35% to the lower-middle income stratum (D+), ensuring socioeconomic representativeness of the municipality.

The original questionnaire consisted of 24 closed-ended questions covering water consumption patterns, perceptions of tap water quality, and household and respondent characteristics. For the purposes of the present study, only a subset of seven questions was used, focusing on subjective self-reported perceptions of tap water odor, perceived health risk from drinking tap water, and key socioeconomic controls. While the survey was originally designed to study willingness to pay for improvements in water quality, the present analysis focuses on perceptual variables relevant to health risk assessment. Further details on the original survey design are reported in Briseño and Macedo [25].

To determine the association between perceived risk of waterborne diseases and perceived unpleasant tap water odor among households in Zapopan, two key perception variables were used in the present analysis. The first item asked: “To what extent do you agree that the tap water in your home has an unpleasant odor?” This question captures a sensory perception of tap water quality and was recoded as a binary variable for this study (1 = Agree, 0 = Neutral or Disagree), labeled "perceived unpleasant odor of tap water". The second item asked: “How worried are you or someone in your family about getting sick from drinking tap water?” which reflects a cognitive–emotional perception of health risk. This variable was also recoded into a binary format (1 = worried, 0 = not worried) in this study. These two self-reported variables therefore represent subjective perceptions (cognitive and sensory, respectively) consistent with prior research on environmental and health risk perception [10, 32].

The rest of the determinants were controlling variables following the conceptual framework of environmental risk perception and social determinants of health. These factors or determinants were chosen according to the previous literature and, attending also to data availability. These variables are in a dummy format except age which is continuous. We simplified such variables following previous environmental risk perception studies, where dichotomous coding facilitates logistic regression interpretation and comparability across studies. For example, education variable was transformed into two categories (those with basic education and those with at least truncated high school). To see the description and descriptive statistics of the variables, please see Table 1.

**Table 1:** Description and descriptive statistics of water-related disease risk perception, perceived unpleasant odor of tap water, and control variables among households in Zapopan, Jalisco, Mexico, October 2018 (household survey)

Name/label	Variable description (type)	Number of observations (dummy values)	Percentage of total
Worried about getting sick	How worried are you or someone in your family about getting sick from drinking tap water? (dummy)	Worried = 315 (1)	Worried = 78.75%
		Neutral or Not worried = 85 (0)	Neutral or Not worried = 21.25%
Perceived unpleasant odor	To what extent do you agree that tap water has an unpleasant odor? (dummy)	Agree = 199 (1)	Agree = 49.8%
		Neutral or Disagree = 201 (0)	Neutral or Disagree = 50.2%
*Controlling variables*			
Medium_high income	Monthly household income:	Medium or high = 240 (1)	Medium or high = 60%
	1. $2,650 to $13,254 (low income)	Low = 160 (0)	Low = 40%
	2. $13,254 (medium/high income) (dummy)		
Boiled_water	Do you boil your water? (dummy)	Boiled = 379 (1)	Boiled = 94%
		Not boiled = 21 (0)	Not boiled = 6%
Home_ownership	What type of accommodation do you live in? (dummy)	Own house = 296 (1)	Own house = 74%
		Other = 104 (0)	Other = 26%
Age	Continuous variable	Mean = 45.51	–
		Std. Dev. = 19.09	
At least high school^a^	Education Levels:	Basic = 100 (0)	Basic = 25%
	1. Basic education	At least truncated High school = 300 (1)	At least truncated High school = 75%
	2. At least truncated High school (it includes complete degree and postgraduate) (dummy)		

Source: Own elaboration based on Briseño, H., & Macedo, E. C. [25]

^a^Education variable was transformed and recorded as binary. Note: Values in parentheses indicate the coding used in the statistical model (1 = Yes, 0 = No)

### Statistical analysis

In order to model the structure of the survey data, the potential association between the perception of waterborne diseases and perceived unpleasant odor of tap water, and other determinants, the current analysis used a logit binomial model approach. For modelling the probability of having a waterborne disease or not, this study assumes a binomial distribution [33],

$$\begin{aligned} y_i \sim \text {Binomial}(\mu _i). \end{aligned}$$

In this case, $\mu _i$ is a binomial variable with values of 1 when the individual *i* was worried about having a disease from drinking tap water, or 0 when an individual *i* did not perceive a risk of waterborne disease from drinking tap water. According to our data, the association of perceived risk of waterborne disease and perceived tap water quality (controlling for other drivers) can be modelled as:

$$\begin{aligned} \mu _{it} & = \alpha + \beta _1 \,\text {Perceived unpleasant odor}_{i}\\ & \quad + \beta _2 \,\text {Medium\_high income}_{i} + \beta _3 \,\text {Boiled\_water}_{i}\\ & \quad + \beta _4 \,\text {Home ownership}_{i} + \beta _5 \,\text {Age}_{i}\\ & \quad + \beta _6 \,\text {At least high school}_{i} + \varepsilon _{i} \end{aligned}$$

where $\text {Perceived unpleasant odor}_i$ denotes our covariate of interest, perceived unpleasant odor of tap water for individual *i*. Meanwhile, $\text {Medium\_high income}_i$ denotes that individual *i* has a medium or high monthly salary. $\text {BoiledWater}_i$ identifies individuals *i* who boil their tap water. Similarly, $\text {HomeOwnership}_i$ represents individuals *i* who own the house or dwelling in which they live. $\text {Age}_i$ corresponds to the age of individual *i*. $\text {At least high school}_i$ captures the individuals *i* with at least truncated high school education. Lastly, $\varepsilon _{i}$ is the error term.

We analyzed the potential overdispersion of the data, running three models: binomial, quasibinomial, and negative binomial. To select the proper model, we analyzed the ratio of the residual deviance to the degrees of freedom in the binomial (logit) model; a value lower than 1.5 indicates no evidence of overdispersion. The quasibinomial and negative binomial models were considered as more flexible alternatives, as they include additional parameters to account for overdispersion—$\phi$ in the quasibinomial model and $\theta$ in the negative binomial model—while the standard binomial model assumes that the variance is entirely determined by the mean.To evaluate whether this added complexity was statistically justified, we applied the Likelihood Ratio Test (LRT) to compare the binomial and quasibinomial models, given that the binomial is a restricted case of the quasibinomial when $\phi$ = 1. A non-significant LRT ($p> 0.05$) suggests that the simpler binomial model fits the data equally well without requiring an extra dispersion parameter. For comparisons involving the negative binomial model—which is not nested within the binomial—we relied on the Akaike Information Criterion (AIC) and the ratio of residual deviance to degrees of freedom, where lower AIC values and deviance ratios below 1.5 denote better model performance. We used R software [34] for the analysis of the data. The models were executing with the glm and glm.nb R codes for the binomial, quasibinomial, and negative binomial specifications, respectively.

## Results

### Descriptive analysis

Table 1 presents an overview of the description and descriptive statistics of the used variables in this study (water diseases, perceived odor in tap water, and controlling variables) in Zapopan city, Jalisco, Mexico. From the total number of the interviewed people in the sample, 78.7% of them reported to be worried or someone in their family about having a disease due to drink tap water. Therefore, the majority of the individuals perceived risk of waterborne disease for drinking tap water. Meanwhile, half of the interviewed people, 49.8%, perceived bad tap water quality (smells bad) in their homes. Less than half of the interviewed individuals had lower salaries, 40%; the rest declared to perceived medium and higher salaries, 60%. A quarter of the population, 25%, in the sample had basic level of education. Meanwhile, 75% of the people had at least truncated high school degree; it includes complete degree and postgraduate. Around three quarters of the population on the sample, 74%, owner their house. The survey reported that the majority of the individuals, 94% had the habit to boil the water. Finally, the average of the age of the individuals were 45 years old.

### Role of perceived unpleasant odor of tap water on perceived risk of waterborne disease

The odds ratio estimates from the logistic model, which assess the association between the perceived risk of waterborne disease and the perceived unpleasant odor of tap water while controlling for socioeconomic factors (income, education, and home ownership), age, and boiling practices are presented in Table 2. The coefficient for the variable representing perceived unpleasant odor was positive and statistically significant, indicating that individuals who perceive an unpleasant odor in their tap water are more likely to consider tap water consumption as a potential health risk. Specifically, the odds ratio associated with unpleasant odor was 1.29, suggesting that, with all other factors constant, people who report that their tap water has an unpleasant odor have approximately 30% higher odds of perceiving a risk of waterborne disease compared to those who do not perceive such odor. This result highlights the influence of sensory cues—particularly the perception of odor—on individual assessments of health risks associated with drinking tap water. The significance of this association indicates that, even in the absence of objective evidence of contamination, sensory perception constitutes a consistent predictor of perceived risk of waterborne disease.

**Table 2:** Association between perceived unpleasant odor of tap water and perceived health risk from drinking tap water among households in Zapopan, Jalisco, Mexico, October 2018 (household survey). Odds ratio estimates from logistic regression models

	Binomial		Quasibinomial		Negative binomial	
Variable	OR	95% CI	OR	95% CI	OR	95% CI
(Intercept)	2.47	(0.09–68.62)	2.47	(0.09–70.99)	0.72	(0.04–3.59)
Perceived unpleasant odor (1 = yes)	1.30	(1.14–1.48)	1.30	(1.14–1.48)	1.12	(1.03–1.22)
Medium-high income (1 = yes)	0.69	(0.45–1.07)	0.69	(0.45–1.07)	0.86	(0.64–1.13)
Boiled water (1 = yes)	0.68	(0.03–17.52)	0.68	(0.03–18.12)	0.83	(0.18–14.71)
Home ownership (1 = yes)	0.76	(0.45–1.28)	0.76	(0.45–1.29)	0.90	(0.66–1.25)
Age (years)	0.99	(0.98–1.00)	0.99	(0.98–1.00)	1.00	(0.99–1.00)
At least high school (1 = yes)	0.62	(0.39–0.97)	0.62	(0.39–0.98)	0.82	(0.61–1.10)
AIC	509.10		NA^*a*^		683.30	

Results are reported as odds ratios (OR) with 95% confidence intervals (CI). Confidence intervals that do not include 1 indicate statistically significant associations at the 5% level. All models control for income, education, age, home ownership, and water-boiling practices.

^*a*^AIC not available for the quasibinomial model (quasi-likelihood has no defined log-likelihood). Note that for each dummy variable the reference category is illustrated with the value = 0 in Table 1. For example, the reference category for “At least high school” is “Basic education”.

With respect to the socioeconomic determinants, the education variable and income were found significant (at the 95% and at the 90%, respectively) and negatively associated with the response variable. Their odds ratio associated with getting sick for drinking water were 0.62 and 0.69, respectively. Meaning that if a person with at least truncated high school (compared to those with basic education) or with medium or higher monthly salary (compared to those with a lower salary), were less likely to perceive a risk of waterborne disease from drinking tap water. Specifically, the perception of health risk decreased by approximately 38% among respondents with at least truncated high school and by 31% among those in higher and medium income categories. These results suggest that socioeconomic advantages are linked to a lower perceived vulnerability to water-related illness. In other words, individuals with higher income or education levels tend to feel less at risk because they can adopt preventive measures, such as using water filters or consuming bottled water, which may reduce exposure or perceived risk. The home ownership variable was also negatively associated with perceived risk (odds ratio = 0.76), although this relationship was not statistically significant. This suggests that individuals living in their own homes, compared to those individuals with not owner their homes, may experience slightly lower concern about getting sick from tap water. For the rest of controlling variables, age and boiled water; the results illustrate that the age variable was significant (at the 95%) and negatively associated with perceived risk for drinking water, its odds ratio was 0.98. This implies that for each additional year of age, the odds of perceiving a health risk from drinking tap water decreased by about 2%, keeping all other variables constant. Older respondents may have adapted to local water conditions over time, perceiving less risk. In contrast, the boiled water variable, although negatively associated (odds ratio = 0.67), did not reach statistical significance, suggesting no clear relationship between the practice of boiling water and the perception of getting sick from drinking tap water.

After analyzing the deviation of the binomial, quasibinomial, and negative binomial models, we chose the binomial model as it did not present any trace of overdispersion. The overdispersion ratio —computed as the residual deviance divided by the degrees of freedom— was found to be less than 1.5. This confirms that the binomial (logit) model adequately captured the data variability. The Likelihood Ratio Test (LRT) comparing the binomial and quasibinomial models yielded a non-significant result, indicating that the inclusion of an additional dispersion parameter did not significantly improve model fit. Moreover, the binomial model exhibited the lowest Akaike Information Criterion (AIC) value among the three models, reinforcing its parsimony and superior overall fit. Taken together, the evidence from the deviance ratio, AIC, and LRT consistently supports the binomial model as the most statistically robust and efficient choice. Therefore, the binomial (logit) model, presented in Table 2, was retained as the final specification for the analysis.

## Discussion

This study has explored the association of subjective perceptions of unpleasant odor in tap water and perceived health risks from waterborne diseases in Mexico (Zapopan, Jalisco), controlling for different health drivers. To the best of our knowledge this is the first work in this area that aims to account such association. Specifically, our findings revealed that the perception of a unpleasant odor in tap water increases the odds of perceiving a health risk by 30% (OR = 1.29). controlling for socioeconomic and demographic factors. This result underscores how subjective sensory attributes can disproportionately influence health-related behaviors, consistent with previous studies showing that perceived odor, taste, or color of tap water are closely linked to heightened concerns about waterborne disease and perceived health risks [11, 32, 35].

Our findings are consistent with existing literature that highlights the importance of water quality perceptions and perceived health risk [10, 32, 35, 36]. For instance, [35] showed that negative perceptions of tap water quality in the United States, often triggered by poor taste, odor, and color, were strongly linked to heightened health risk concerns and increased reliance on bottled water. [36] found that low-income and minority households were more likely to distrust tap water and perceive it as unsafe, which contributed to disparities in health outcomes. [10], in a systematic review of WASH studies, demonstrated that perceived water-related health risks motivated protective behaviors such as treatment or source switching, highlighting the perception–health nexus. Similarly, [32] developed and validated a Water Quality Perception Scale (WQPS), confirming that risk perceptions of poor water quality are a significant predictor of health-protective decisions. In the context of the mexican municipality of Zapopan, where concerns about water quality are well-documented [14], our study adds an empirical layer by showing that these perceptions directly translate into a heightened fear of illness. The high proportion of our sample (78%) that expressed concern about waterborne diseases underscores the urgency of addressing this issue.

In addition to sensory perception, our study identifies socioeconomic factors as significant moderators of risk perception. We observed that both education (OR = 0.62) and income (OR = 0.69) were negatively associated with perceived risk. Rather than implying that waterborne disease risk is absent, these results may reflect that individuals with higher income or education levels feel less at risk because they can adopt preventive measures—such as using water filters or consuming bottled water—that reduce exposure or perceived vulnerability. It is also possible that higher socioeconomic status is associated with residential areas served by better water infrastructure or with greater access to reliable information about water safety. These structural and behavioral differences may help explain why perceived health risks are lower among these groups. Our findings are consistent with other studies [37–39]. For instance, [37] showed that individuals with higher education and income had a greater understanding of water contamination and its health implications, with 35% identifying diarrhea as the most prevalent waterborne disease. In contrast, [38] found that 98% of participants in low-income Latino households in Nogales, Arizona feared illness from drinking tap water, and perceived the risk of consuming it as comparable to drinking and driving. Similarly, [39] revealed that maternal education was significantly associated with knowledge of waterborne diseases and prevention practices, while household income was not, underscoring the stronger role of education over income in shaping risk perception and health-related awareness.

Age was also inversely related to perceived health risks, with older individuals reporting lower concern. This may be due to cumulative adaptation to local water conditions, lower perceived susceptibility, or generational differences in attitudes toward public services and water quality. These findings highlight an opportunity for future research to explore how age and generational experiences shape perceptions of water safety. While prior studies have documented age-related differences in protective behaviors, such as the adoption of water filters [40], little is known about how these behavioral patterns correspond to perceived health risks across age groups.

Finally, home ownership and water-boiling practices were negatively associated with perceived risk, but not statistically significant. While not conclusive, these patterns suggest that both structural security (owning a home) and protective behaviors (boiling water) may contribute to a sense of control, thereby moderating risk perception. Prior research in rural China has documented the benefits and trade-offs of boiling water [41], but our findings highlight the importance of exploring these practices within urban Mexican households, where boiling remains widespread despite the availability of bottled water.

Together, these results point to a dual challenge for water governance in Mexico: improving objective water quality while simultaneously addressing public perceptions. The widespread distrust of tap water documented nationally is mirrored in Zapopan, where almost half of the respondents perceived their water to have a bad smell, and more than three-quarters expressed concern about waterborne illnesses [9]. Left unaddressed, these perceptions may perpetuate costly coping strategies, such as the reliance on bottled water, with implications for environmental and social equity [11, 16].

From a policy perspective, the study underscores the need for risk communication strategies that go beyond technical improvements in water infrastructure. Public health messaging should directly address sensory concerns and emphasize evidence-based assessments of water safety, while also considering the socioeconomic and demographic determinants that shape risk perception. Targeted campaigns aimed at younger populations, lower-income households, and those with limited educational attainment could help rebuild trust in municipal water systems. Moreover, integrating community participation in water monitoring and reporting may reduce the gap between perceived and actual water quality, thereby enhancing both health outcomes and public confidence. The municipal water utility SIAPA (Intermunicipal System for Drinking Water and Sewerage Services) is responsible for monitoring drinking water quality in Zapopan in compliance with Mexican regulations, including routine physicochemical and microbiological analyses [42, 43]. Public access to detailed results is limited; however, official reports and national databases maintained by the National Water Commission [44] provide the most reliable publicly available information on water quality and regulatory compliance.

This study is subject to some limitations. The analysis is based on cross-sectional survey data, which precludes causal inference. Longitudinal designs would be valuable to assess how perceptions evolve in response to changes in water quality or health events. The study relies on self-reported measures of perceived quality and risk, which may not align with objective indicators of contamination. The variable used—perceived unpleasant odor of tap water—captures only one sensory dimension of water perception. While odor is a salient cue that often triggers concern about water safety, it does not include other attributes such as taste, color, or clarity. Therefore, this variable should be interpreted as an indicator of perceived odor rather than a comprehensive measure of perceived water quality, and the estimated association between perceived water quality and perceived health risk may be conservative, as respondents could consider tap water unsafe for reasons unrelated to odor. Finally, although the odds ratio for perceived unpleasant odor (OR = 1.29) is modest, it is statistically significant and consistent with recent perception-based studies [12, 32, 35]. Even small changes in odds can reflect meaningful shifts in how individuals assess waterborne health risks, especially when perceptions are influenced by sensory cues rather than objective contamination measures. Despite these potential shortcomings, the used survey presents some advantages. [45] emphasizes that primary data such as survey are real-time, original, and tailored to the precise objectives of the study, whereas secondary data are often historical and may not align with current research needs.

In conclusion, this study demonstrates that perceived odor in tap water is associated with perceived risk of waterborne disease in Zapopan, after controlling for socioeconomic and demographic factors. By highlighting the importance of subjective perceptions, the findings contribute to a deeper understanding of how environmental, social, and behavioral factors intersect in shaping public health outcomes. Addressing both the physical and perceptual dimensions of water quality will be essential to advancing equity-driven strategies for safe drinking water. Additionally, public awareness campaigns could be designed to address these perceptions by providing clear and evidence-based information about tap water quality and by investing in improvements to sensory attributes to increase public trust. In doing so, reliance on less sustainable alternatives like bottled water can be reduced, thereby promoting equity and sustainability in access to clean drinking water in Mexico and comparable contexts globally.

## Data Availability

The survey is property of the Universidad Panamericana. Note that this study received ethical review and approval from the Ethics and Scientific Review Committee of the Universidad Panamericana. All participants, interviewees, were fully informed about the study before providing their consent. Participation was entirely voluntary. The dataset generated during this study are available from the corresponding author upon reasonable request.
